# Role of Neutrophils in the Pathogenesis of Nonalcoholic Steatohepatitis

**DOI:** 10.3389/fendo.2021.751802

**Published:** 2021-10-11

**Authors:** Seonghwan Hwang, Hwayoung Yun, Sungwon Moon, Ye Eun Cho, Bin Gao

**Affiliations:** ^1^ College of Pharmacy and Research Institute for Drug Development, Pusan National University, Busan, South Korea; ^2^ Laboratory of Liver Diseases, National Institute on Alcohol Abuse and Alcoholism, National Institutes of Health, Bethesda, MD, United States

**Keywords:** neutrophil, nonalcoholic steatohepatitis, nonalcoholic fatty liver disease, inflammation, fibrosis

## Abstract

Nonalcoholic fatty liver disease (NAFLD) includes a spectrum of liver disorders, from fatty liver to nonalcoholic steatohepatitis (NASH), cirrhosis, and hepatocellular carcinoma. Compared with fatty liver, NASH is characterized by increased liver injury and inflammation, in which liver-infiltrating immune cells, with neutrophil infiltration as a hallmark of NASH, play a critical role in promoting the progression of fatty liver to NASH. Neutrophils are the first responders to injury and infection in various tissues, establishing the first line of defense through multiple mechanisms such as phagocytosis, cytokine secretion, reactive oxygen species production, and neutrophil extracellular trap formation; however, their roles in the pathogenesis of NASH remain obscure. The current review summarizes the roles of neutrophils that facilitate the progression of fatty liver to NASH and their involvement in inflammation resolution during NASH pathogenesis. The notion that neutrophils are potential therapeutic targets for the treatment of NASH is also discussed.

## 1 Introduction

Neutrophils are the most populous subset of leukocytes in the circulation and participate in various processes of immune reactions and inflammation (1). Neutrophils act as an effector of innate immunity to handle microorganism infection and execute a series of reactions to maintain homeostasis during tissue injury (2–4). Neutrophils are equipped with a variety of protective mechanisms against infection. These include phagocytosis, reactive oxygen species (ROS) production, oxidative burst, release of proteolytic enzymes *via* degranulation, and formation of web-like structures called neutrophil extracellular traps (NETs) that extrude genomic DNA and enzymes to fight against microorganisms (5). With regard to sterile inflammation, neutrophils are also activated and recruited to the site of injury as one of the first responders and participate in the inflammatory response to restore the physiological function of the tissue (3, 4). Because of their versatile functions, neutrophils have been highlighted as a critical mediator of diseases in multiple organs, including the liver.

**Figure 1:**
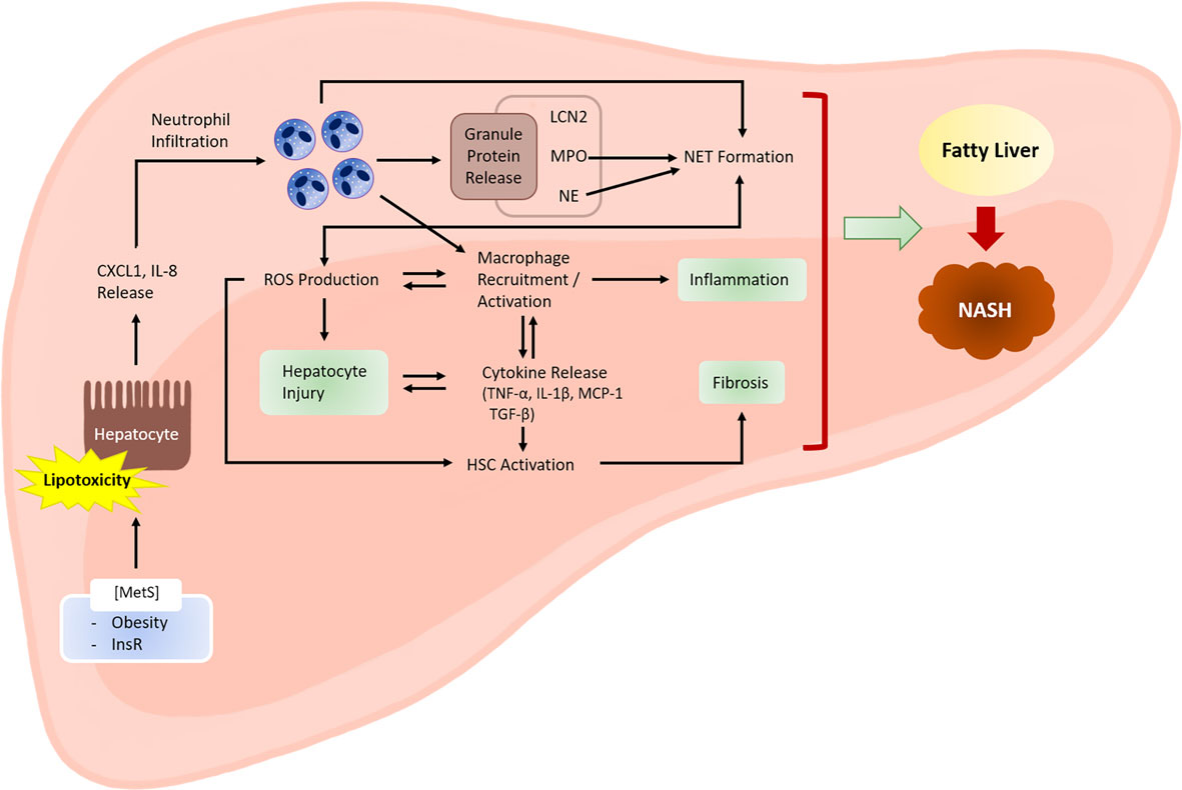
Role of neutrophils in the development of NASH. Metabolic syndrome is often associated with excessive lipid accumulation in the liver. Under these conditions, the probability of hepatocytes being exposed to lipotoxic lipid species, such as fatty acids, ceramides, cholesterol, and sphingolipids, is high. Lipotoxic hepatocytes release neutrophil-recruiting chemokines, including CXCL1 and IL-8. Infiltrating neutrophils exert various actions that facilitate NASH development. Activated neutrophils produce ROS *via* an oxidative burst that involves the activity of enzymes, such as NADPH oxidase 2. ROS may directly cause hepatocyte injury. ROS also activate and recruit macrophages, which further enhance hepatocyte injury and inflammation by releasing inflammatory cytokines. Cytokines released by macrophages (e.g., transforming growth factor-β) activate hepatic stellate cells (HSCs) and promote fibrosis. Neutrophil-derived ROS also contribute to HSC activation. Neutrophil granule proteins, such as LCN2, MPO, and NE, have increasingly been recognized to contribute to sterile inflammation, although the exact mechanisms through which they contribute to NASH are unclear. NETs mediate inflammation during NASH development through mechanisms that are not yet fully understood. Neutrophil-derived factors such as ROS and granule proteins (e.g., MPO and NE) contribute to NET formation. Hepatocyte injury, inflammation, and fibrosis are the three hallmarks of NASH. MetS, metabolic syndrome; InsR, insulin resistance.

Although hepatitis C virus (HCV) and hepatitis B virus (HBV) infections have traditionally been the leading causes of chronic liver disease that requires liver transplantation, the position of HCV and HBV infection has recently been challenged by several other etiologies, among which is nonalcoholic steatohepatitis (NASH) whose cases are rapidly increasing (6). NASH has recently become prevalent: fatty liver is observed in approximately 25% of the adult population, and 25% of the individuals with fatty liver are estimated to proceed to NASH (7). In the USA, NASH has become the second most common indication for liver transplantation (8, 9). NASH is marked by liver inflammation and damage caused by fat accumulation in the liver (10). The inflammatory properties of NASH are closely associated with the recruitment of innate immune cells, including neutrophils and monocytes; thus, investigators have recently focused on the role of neutrophils in exacerbating inflammation and tissue damage during NASH development (11). In this review, we summarize the recent advances in our understanding of the pathological role of neutrophils in the development of NASH (**Figure 1**), as well as their potential as therapeutic targets. The current review also discusses the protective function of neutrophils in NASH pathogenesis, which has recently gained attention (12).

## 2 Pathogenesis of NASH

Nonalcoholic fatty liver disease (NAFLD) encompasses a disease spectrum that ranges from fatty liver to NASH, cirrhosis, and hepatocellular carcinoma (13). Fatty liver is the benign and reversible stage of NAFLD and is caused by excessive fat accumulation in hepatocytes (defined as the presence of lipid droplets in >5% of hepatocytes) (14). NASH is the more severe form of fatty liver and is characterized by the presence of liver injury, inflammation, and fibrosis (15), which are not remarkably observed in the fatty liver of obese humans and mice fed with a high-fat diet (HFD) (16). The mechanism underlying the progression of fatty liver to NASH has been extensively investigated to identify a therapeutic target for the pharmacological intervention of NASH; however, no medications have been approved for use in the treatment of NASH (17). Because this review focuses on the role of neutrophils in NASH pathogenesis, this section highlights the inflammatory basis of the pathogenic events in NASH development and the link between lipotoxicity, hepatocyte injury, and inflammation.

### 2.1 Hepatic Fat Accumulation and Lipotoxicity

Fat accumulation in the liver is a priming factor in the development of NAFLD (18). This event is accompanied by the conversion of surplus energy sources such as carbohydrates and fatty acids into triglycerides, which are stored in the liver as lipid droplets (19). Hepatic fatty acids can come from a variety of sources: i) adipocyte-derived fatty acids generated by lipolysis of triglycerides, ii) *de novo* lipogenesis from sugars (e.g., glucose and fructose), and iii) intake of dietary sugar and lipid species (17). Fatty acid is relatively toxic and thus is preferred to be converted into triglycerides for storage, and mitochondrial fatty acid beta oxidation is another way to dispose of fatty acids (20). However, when there is excessive accumulation of fat in the liver, these protective mechanisms normally fail to completely eliminate fatty acids that can cause cellular stresses in a variety of ways that are discussed in more detail below. In addition, fatty acids serve as a source of several lipotoxic lipid classes (e.g., ceramides and sphingolipids), and they increase in amount during NAFLD progression (21). Free cholesterol is another type of toxic lipid that is elevated in the liver of patients with NASH (22). It is increasingly regarded as a contributing factor to NASH pathogenesis and is widely used as a supplement with NASH-inducing diets in experimental models.

### 2.2 Cellular Stress and Cell Death

Accumulation of lipotoxic lipid species in the liver causes cellular stress in hepatocytes, eventually leading to hepatocyte death (23). At the subcellular organelle level, lipotoxicity induces different types of stress in distinct locations such as the endoplasmic reticulum (ER), lysosomes, and mitochondria (24–26). Among the multiple types of lipotoxic lipids, palmitic acid has been particularly well documented as an inducer of hepatocyte stress. Palmitic acid is increased in the liver of NASH patients and experimental NASH models (27–29), and it triggers ER stress (30). Under the conditions with chronic ER stress, the unfolded protein response may facilitate inflammation and the death of hepatocytes (31). Exposure of hepatocytes to palmitic acid also disrupts mitochondrial and lysosomal functions (32). Severe injury in hepatocytes upon exposure to palmitic acid readily provokes hepatocyte death through the mechanisms involving molecular players that mediate organelle stress. For example, palmitic acid stimulates apoptosis of hepatocytes *via* the extrinsic and intrinsic pathways. Mitochondrial dysfunction and cytochrome c release are critical elements that activate the intrinsic apoptosis pathway (33, 34). Hepatocytes express death receptors such as tumor necrosis factor (TNF)-related apoptosis-inducing ligand (TRAIL) receptors that mediate apoptosis, especially *via* the extrinsic pathway, and the expression of these receptors is increased in NASH patients and mouse models (35). There are also other types of cell death, such as necrosis and pyroptosis, that can be observed in lipotoxic hepatocytes (36–38).

Lipotoxic hepatocytes undergoing cell death release chemokines and cytokines, which recruit and activate the innate immune cells, such as neutrophils and macrophages, for the initiation and amplification of inflammation (39–41). These inflammatory cells also release factors that signal through death receptors on hepatocytes (e.g., TRAIL-R1, TRAIL-R2, TNFR1, and Fas), which may further stimulate hepatocyte death and inflammation, thereby creating a positive feedback loop (36).

### 2.3 Gut Dysfunction

In addition to hepatocyte stress and death, other factors may also enhance inflammation in the pathogenesis of NASH. For example, gut barrier dysfunction is profound in NASH, which is believed to cause the translocation of gut bacteria to the circulation and further to the liver *via* portal vein (42). The compromise in the integrity of intestinal epithelial barrier may also promote the migration of the proinflammatory bacterial products such as endotoxin to the circulation and cause endotoxemia (43). Under endotoxic conditions, the influx of the proinflammatory bacterial products such as lipopolysaccharides to the liver is enhanced and trigger hepatic inflammation and NASH development.

Mouries et al., demonstrated that intestinal epithelial barrier and gut vascular barrier were disrupted in mice with diet-induced NASH (44). Mice with defective intestinal epithelial barrier develop more severe NASH when fed a diet high in fat, fructose, and cholesterol (45). The intestines of NAFLD patients are characterized by the disruption of intestinal tight junction and an increase in cytokine secretion and intestinal inflammation (46).

The diversity of microbial species become altered in NASH patients. Jiang et al. demonstrated that *Escherichia, Anaerobacter, Lactobacillus*, and *Streptococcus* were more abundant in the gut microbiota of NAFLD patients, while *Alistipes* and *Prevotella* were less abundant in NAFLD patients compared to healthy individuals (46). The diversity of microbial species is important for the integrity of the intestinal epithelial barrier and the regulation of metabolic functions; thus the change in their diversity may also contribute to the development of NAFLD and NASH (46, 47).

### 2.4 Inflammation

The progression of fatty liver to NASH is driven by innate immunity, where liver-resident macrophages (also known as Kupffer cells), infiltrating monocytes, and neutrophils play a critical role (48, 49). In the early phase of liver injury, Kupffer cells are activated and release chemokines and proinflammatory cytokines, including C-C motif chemokine ligand 2 and TNF-α, which result in the recruitment of monocytes and neutrophils into the injured liver (50, 51). Interleukin (IL)-1β and IL-6 released by Kupffer cells can further enhance NASH pathogenesis by increasing the lipogenesis and insulin sensitivity in hepatocytes (52–54). The recruited monocytes express high levels of lymphocyte antigen 6C (defined as LY6C^hi^ monocytes) and differentiate into M1 type macrophages that exacerbate inflammation by releasing cytokines and ROS (55–59). Some of these cytokines, including transforming growth factor-β, may also directly act on hepatic stellate cells (HSCs), causing their transformation into myofibroblasts that promote fibrogenesis (60, 61). Different types of macrophages also play an important role in inflammation resolution, which are referred to as either alternatively-activated macrophages or M2 type macrophages. M2 macrophages are known to counteract the proinflammatory environment and participate in tissue repair (62); however, they also promote fibrotic changes in the liver that occur during the pathogenesis of NASH (63, 64).

The advent of single-cell analysis has expanded our understanding of the diverse subsets of infiltrating monocytes and macrophages in the liver, which can be characterized by the expression of distinct surface marker proteins. More recent studies reported that a specific subset of macrophages called NASH-associated macrophages, which express unique marker proteins, such as triggering receptor expressed on myeloid cells 2 (TREM2) and CD9, are enriched in the livers of NASH mouse models and patients (65, 66). Remmerie et al. demonstrated a reduction in Kupffer cells and an increase in a unique population of macrophages expressing SPP1 (also known as osteopontin) in the liver during NASH progression (67). The group of Philippe Gual reported that CD44 which is expressed in macrophages is upregulated during NASH progression and enhances NASH progression by controlling macrophage polarization and infiltration (68). However, whether and how these specific macrophage subsets affect the development of NASH are not fully understood.

Other types of immune cells that contribute to the pathogenesis of NASH include cytotoxic T cells, B cells, T-helper cells (e.g., Th1, Th2, and Th17), dendritic cells, natural killer cells, and innate lymphoid cells, and there is accumulating evidence supporting the pathogenic roles of these immune cells (69, 70). However, the overall pathogenic mechanisms of NASH encompass a broad range of biological events and molecular players, and thus are beyond the scope of the current review. Building upon the information stated in this section regarding the inflammatory aspects of NASH pathogenesis, primarily involving the action of macrophages, the following section will elaborate on the role of neutrophils in NASH development.

## 3 Involvement of Neutrophils in NASH Pathogenesis

Neutrophils are the first type of immune cells that respond to inflammatory changes in various tissues, including the liver, and execute a program that eventually induces a chronic inflammatory state by promoting macrophage recruitment and interacting with antigen-presenting cells (71–73). Thus, neutrophils have been studied as crucial players in the development of inflammatory liver diseases, including NASH. Treatment with a monoclonal antibody against Ly6G, which is present on neutrophils, results in a partial depletion of neutrophils in mice and thus has been used to investigate the role of neutrophils in the development of a variety of diseases (74, 75). Depletion of neutrophils in HFD-fed mice using Ly6G monoclonal antibody treatment significantly reduced the body weight gain, blood glucose levels, and hepatic triglyceride accumulation (76). It also repressed the expression of inflammatory and fibrotic proteins in the liver and decreased the activity of transaminases (e.g., alanine aminotransferase and aspartate aminotransferase), indicating that neutrophils are implicated in metabolic dysregulation, inflammation, and fibrosis during NASH development (76). The neutrophil-to-lymphocyte ratio (NLR) is closely related to the severity of NAFLD. In particular, NLR was correlated with the degree of hepatocyte ballooning, lobular inflammation, and fibrosis in a study of NASH patients (77). A more recent study also reported that NLR was associated with NAFLD activity score, hepatocyte degeneration, steatosis, inflammation, and fibrosis, indicating the possibility that NLR could function as a marker for histological grade and fibrosis stage of NASH (78). Although these studies emphasize the correlation between the abundance of neutrophils and the severity of NASH, there has been a recent increase in our understanding of the mechanism by which hepatic neutrophil infiltration accelerates NAFLD progression, which is further discussed below.

Neutrophil infiltration in the liver is a salient feature of alcoholic steatohepatitis (ASH) and NASH (79), both of which are regarded as the leading causes of end-stage liver disease and liver transplantation (6). In the context of ASH pathogenesis, the mechanisms by which neutrophil population is increased in the circulation, how neutrophils undergo hepatic infiltration, and how these neutrophils are implicated in the exacerbation of liver injury and inflammation have been well-documented (79, 80). In particular, multiple studies have investigated the pathogenic roles of neutrophils in murine models of ASH, such as chronic and binge ethanol feeding (the National Institute on Alcohol Abuse and Alcoholism model) (81–85). Neutrophils produce and release ROS, proteases, and inflammatory mediators to enhance hepatocyte injury and subsequent inflammation and fibrosis (80).

Although the histopathological features of NASH and ASH are similar to each other, albeit not identical, their immunological pathogenic mechanisms are different (86). For example, lipotoxicity-induced immune cell activation is regarded important to NASH pathogenesis, while the concept of lipotoxicity has not been highlighted in the field of ASH research although fatty liver stage is involved in the spectrum of alcoholic liver disease. Thus, it is not recommended to simply apply the knowledge obtained from ASH research to the understanding of NASH pathogenesis and the identification of NASH therapeutic targets. Compared with the well-documented role of neutrophil infiltration in the development of ASH, the contribution of neutrophils to NASH development has been relatively obscure, in part because of the lack of appropriate animal models that can recapitulate the NASH-associated elevation in hepatic neutrophil population as well as molecular features corresponding to the activation and recruitment of neutrophils.

### 3.1 HFD^+CXCL1^-Induced NASH Mouse Model With Increased Hepatic Neutrophil Infiltration

Diet-induced obesity is a widely used model to study fatty liver in mice; however, it is generally difficult to embody a NASH-like environment in the liver of mice by simply feeding them with an HFD chronically. Obesity, diabetes, and hyperlipidemia are major risk factors for NAFLD in humans, and long-term HFD feeding in mice gives rise to these risk factors. However, long-term HFD feeding seldom induces hepatic inflammation or hepatic infiltration of neutrophils (82).

Inflammation is a key event that promotes the progression of fatty liver to NASH, and the inflammation observed in NASH patients is characterized by a significant infiltration of neutrophils in the liver and other immune cells such as macrophages (61, 87). NASH-associated neutrophil infiltration in the liver is often accompanied by elevated expression of chemokines for neutrophil chemotaxis. Bertola et al. reported that NASH patients had a higher hepatic expression of chemokines that recruit neutrophils, such as C-X-C motif chemokine ligand 1 (CXCL1) and IL-8, than obese individuals with fatty liver (88).

Recent studies have revealed several differences in neutrophil biology between mice and humans, which may confer resistance to fatty liver to NASH progression in mice. First, mice have relatively fewer neutrophils in the circulation (~1 × 10^9^/L) than humans (~4 × 10^9^/L) (1, 85). In addition, inflamed mouse hepatocytes have less ability to attract neutrophils than inflamed human hepatocytes as the human *IL-8* gene has no counterpart in mice, and mouse hepatocytes induce CXCL1 less effectively than human hepatocytes when exposed to inflammatory mediators (89). Accordingly, metabolic dysregulation and injury in the mouse liver less effectively recruit neutrophils, which could partially explain why HFD-fed mice were more likely to develop fatty liver than NASH.

Although there is no experimental model that reflects the full spectrum of NAFLD progression in mice (90), several dietary NASH models have recently been established by adding lipotoxic materials (e.g., cholesterol) and carbohydrates to a regular HFD (90). Some of these NASH models have been reported to show increased neutrophil infiltration in the liver (91–93).

Experimental NASH mouse models have been mostly induced by dietary compositions that provide overnutrition and contribute to liver injury; however, recent studies have reported new approaches that exploit the molecular mechanisms that mediate hepatic neutrophil infiltration and thus accelerate the progression of fatty liver to NASH. Adenovirus-driven overexpression of *Cxcl1* in 3-month HFD-fed mice enhanced the expression of neutrophil-recruiting chemokines in hepatocytes and promoted the recruitment of neutrophils in the liver (16, 89). CXCL1-driven neutrophil infiltration promotes ROS production and activates stress kinases, including apoptosis signal-regulating kinase 1 and p38 mitogen-activated protein kinase (p38MAPK), which relay oxidative stress to cell death signaling (94). ROS also impair the proper folding of proteins, resulting in ER stress, which is often observed in NASH patients (31, 95, 96). CXCL1-induced, neutrophil-driven liver damage further led to inflammatory and fibrogenic processes and facilitated the progression of NASH; the expression profiles of the genes involved in inflammation and fibrosis were similar to those found in NASH patients (16). The overexpression of the human *IL-8* gene in mice could also increase hepatic neutrophil infiltration and facilitate the progression of fatty liver to NASH; the concomitant overexpression of *Cxcl1* and *IL-8* further amplified the effect of the single overexpression of either *Cxcl1* or *IL-8* (89).

Neutrophilic oxidative bursts are crucial for ROS production by activated neutrophils (5). CXCL1-induced liver injury in HFD-fed mice was found to be dependent on neutrophil cytosolic factor 1 (also known as p47phox), which is one of the components of the NADPH oxidase 2 complex that mediates oxidative burst (97, 98), corroborating the importance of neutrophilic ROS production in neutrophil-driven NASH development (16). In addition, CXCL1-induced NASH was ameliorated by treatment with IL-22, which is a cytokine produced by immune cells such as Th17, Th22, and type 3 innate lymphoid cells (ILC3s) (99) but acts on epithelial cells, including hepatocytes, by upregulating various genes with cytoprotective and anti-oxidative properties (100, 101). In particular, the protective effect of IL-22 against neutrophil-driven NASH was reversed in mice lacking the genes encoding the two antioxidant enzymes, namely, metallothionein-1 and metallothionein-2, which implies the important function of ROS in neutrophil-driven NASH development. Interestingly, neutrophil elastase (NE) or NET was not crucial in CXCL1-induced NASH development because deletion of the gene encoding NE or peptidyl arginine deiminase-4 (PAD4) failed to reverse the NASH-inducing ability of CXCL1 overexpression in HFD-fed mice (16).

CXCL6 is another chemokine that recruits neutrophils by binding CXCR1 and CXCR2 (102). Due to the similar function of CXCL6 to that of CXCL1 and IL-8, it is reasonable to speculate that CXCL6 might be involved in the pathogenesis of NASH. However, the role of CXCL6 in NASH pathogenesis is still obscure. For neutrophils to be recruited from the blood to the affected sites, neutrophils interact with adhesion molecules such as E-selectin expressed on endothelial cells. E-selectin has been reported to be upregulated in the liver of NASH patients compared with the fatty liver (88). In CXCL1-induced NASH model, the expression of E-selectin was also highly elevated in the liver, indicating the involvement of E-selectin in the development of NASH in mice (16).

### 3.2 Role of Neutrophil-Specific MicroRNA-223 in NASH Development

miRs have emerged as important regulators of various genes involved in metabolism and inflammation in the liver (103–105). Several miRs, including miR-122, miR-192, miR-223, miR-21, and miR-29, affect the pathogenesis of NAFLD *in vitro* and in experimental animal models (106–113). These miRs usually have specific cell types, where they show the highest expression. For example, miR-122 and miR-192 are highly expressed in hepatocytes, miR-21 is commonly found in the circulation, and miR-29 is enriched in HSCs (114). MiR-223 is particularly interesting and is expressed at the highest levels in neutrophils, whose activation and maturation are repressed by miR-223 (115–117). Although miR-223 is also expressed in macrophages and is involved in macrophage polarization, its expression levels in macrophages are approximately 10% that in neutrophils (118); thus, it is regarded as a neutrophil-specific miR (119). miR-223 is upregulated in hepatocytes in HFD-fed mice and NASH patients (107, 109). miR-223 targets and inhibits several genes in mice that are involved in inflammation and tumorigenesis, such as C-X-C motif chemokine ligand 10 (CXCL10) and transcriptional coactivator with PDZ-binding motif (TAZ), and deletion of the miR-223 gene in HFD-fed mice promoted the development of NASH and NASH-associated hepatocellular carcinoma (109). Mechanistically, He et al. reported that miR-223, which is abundantly expressed in neutrophils, was transported to hepatocytes through the extracellular vesicles (EVs) derived from neutrophils (118). In particular, the transfer of miR-223-enriched EVs is mediated by the interaction between low-density lipoprotein receptor (LDLR) in hepatocytes and apolipoprotein E (APOE) in EVs (118). In addition, miR-223 protects against liver fibrosis by targeting multiple genes in hepatocytes and HSCs (120), which may contribute to the role of miR-223 in preventing NASH progression. The IL-6 signaling in myeloid cells, such as neutrophils, is important for the generation and release of miR-223-enriched EVs, which may inhibit the progression of fibrosis in NASH-associated fibrosis (121, 122). As miR-223 is expressed by both hepatocytes and neutrophils, specific deletion of miR-223 in each cell population by crossing miR-223-floxed mice with either Albumin Cre or Lysozyme M Cre mice will help better understand its role in the pathogenesis of NASH.

Elevation of miR-223 levels in the circulation and liver in NASH patients may be a compensatory mechanism to modulate the proinflammatory environment that prevails during NAFLD progression; thus, the deletion of miR-223 gene could accelerate NASH development in HFD-fed mice. Along with the model that is characterized by an increase in neutrophil infiltration in the liver, inhibition of the anti-inflammatory function of neutrophilic miR-223 could be another method to develop a genetic NASH model (85, 116, 117, 123).

### 3.3 Role of Neutrophil-Derived Specific Molecules in NASH Development

Neutrophil granules are sites where a large amount of protein is expressed, which mediates various neutrophil functions (124). Neutrophil granule proteins are released into the phagosome or extracellular space by degranulation and execute their functions (125). The different types of granules include azurophilic granules, specific granules, gelatinase granules, and secretory granules (**Table 1**) (126, 127). In particular, emerging evidence has demonstrated that the proteins expressed by azurophil granules [e.g., myeloperoxidase (MPO) and NE] and specific granules [e.g., lipocalin 2 (LCN2)] play a key role in various inflammatory processes during NASH pathogenesis. S100 calcium-binding protein A8 (S100A8, also known as MRP8) and S100 calcium-binding protein A9 (S100A9, also known as MRP14) are Ca^2+^-binding proteins that constitute 40% of neutrophil cytosolic protein weight. Their roles in NASH have also been discussed (128). In addition, neutrophils can produce a large number of inflammatory cytokines, chemokines, and inflammatory mediators, which likely play an important role in controlling NASH development and progression (129). Here, we primarily discussed the role of neutrophil-derived specific molecules in NASH development and progression. The changes in the levels of these molecules in experimental NASH as well as in clinical settings and the consequences of genetic or pharmacologic modulation of these factors in experimental NASH were also summarized (**Tables 2** and **3**).

**Table 1:** Types of neutrophil granules and their contents.

Granule type	Contents
Azurophilic granules	myeloperoxidase, neutrophil elastase, cathepsin G, defensins, proteinase 3, azurocidin, lysozyme
Specific granules	collagenase, gelatinase, lipocalin 2, pentraxin 3, cathelicidin, matrix metalloprotease 8, lactoferrin, haptoglobin, lysozyme, cytochrome b558, CD11b, formyl peptide receptor
Gelatinase granules	Collagenase, acyl transferase, cathepsins, gelatinase
Secretory granules	CD11b, cytochrome b558, alkaline phosphatase, formyl peptide receptor

**Table 2:** Elevation of neutrophil-related factors in NASH patients and experimental NASH models.

	Level change in NASH	Location	NASH induction model	Reference
MPO	↑	Liver and plasma	NASH patients	(130)
	↑	Plasma	NASH patients	(91)
	↑	Liver	MCD-fed mice	(131)
	↑	Liver	*Ldlr*−/− mice fed an HFD	(132)
	↑	Liver	CXCL1 overexpression in HFD-fed mice	(16)
NE	↑ (NE to AAT ratio)	Circulation	NASH patients	(133)
	↑	Liver	Western diet-fed mice	(134)
LCN2	↑	Circulation and hepatic non-parenchymal cell fraction	*Apoe*−/− mice fed an HFHCD	(135)
	↑	Circulation and liver	NASH patients	(135)
	↑	Liver	NASH patients	(136)
	↑	Liver	Mice fed a high-fat, high-sugar diet	(137)
	↑	Liver	FLS mouse model	(138)
NET formation	↑	Liver	STAM mice	(92)
	↑ (MPO-DNA levels)	Serum	NASH patients	(92)
	↑ (citrullinated histone H3)	Liver	NASH patients	(139)
	↑	Liver	Mice fed an MCDHFD	(140)

AAT, alpha-1-antitrypsin; APOE, apolipoprotein E; CXCL1, C-X-C motif chemokine ligand 1; FLS, fatty liver Shionogi; HFD, high-fat diet; HFHCD, high-fat, high-cholesterol diet; LCN2, lipocalin 2; LDLR, low-density lipoprotein receptor; MCD, methionine-choline-deficient diet; MCDHFD, methionine-choline-deficient high-fat diet; MPO, myeloperoxidase; NASH, nonalcoholic steatohepatitis; NE, neutrophil elastase; NET, neutrophil extracellular trap; STAM, Stelic animal model of NASH.

**Table 3:** Modulation of neutrophil-related factors that affect the degree of experimental NASH.

	Modulation method	NASH induction model	Effect on NASH pathology	Reference
MPO	Deletion of the gene encoding MPO	HFHCD feeding in mice	↓ liver injury and liver fibrosis	(91)
	Pharmacological inhibition of MPO by AZM198 treatment	HFHCD feeding in mice	↓ liver injury and liver fibrosis	(91)
NE	Deletion of the gene encoding NE	HFD feeding in mice	↓ hepatic lipid content and inflammation	(141)
	Deletion of the gene encoding NE	Western diet feeding in mice	↓ steatosis and liver inflammation	(134)
	NE inhibition by sivelestat	HFHCD feeding in *Apoe*−/− mice	↓ steatosis, liver injury, inflammation, and NASH score	(142)
LCN2	Deletion of the gene encoding LCN2	HFHCD feeding in *Apoe*−/− mice	↓ liver injury, inflammation, and NASH score	(135)
	Treatment of recombinant LCN2	HFHCD feeding in *Apoe*−/− mice	↑ liver injury, inflammation, and NASH score	(135)
NET formation	DNase treatment	STAM mice	↓ hepatic macrophage infiltration, liver inflammation, and NASH activity score	(92)
	Deletion of the gene encoding PAD4	STAM mice	↓ hepatic macrophage infiltration, liver inflammation, and NASH activity score	(92)
	DNase treatment	MCDHFD feeding in mice	↓ liver injury, inflammation, and fibrosis	(140)
CXCL1, IL-8	Overexpression of CXCL1 and/or IL-8	HFD feeding in mice	↑ liver injury, inflammation, and fibrosis	(16, 89)

APOE, apolipoprotein E; CXCL1, C-X-C motif chemokine ligand 1; HFD, high-fat diet; HFHCD, high-fat, high-cholesterol diet; IL-8, interleukin-8; LCN2, lipocalin 2; MCDHFD, methionine-choline-deficient high-fat diet; MPO, myeloperoxidase; NASH; nonalcoholic steatohepatitis; NE, neutrophil elastase; NET, neutrophil extracellular trap; PAD4, peptidyl arginine deiminase-4; STAM, Stelic animal model of NASH.

#### 3.3.1 Myeloperoxidase

Myeloperoxidase (MPO) catalyzes the generation of ROS, which is crucial for the ability of neutrophils to kill microorganisms (5). ROS production by MPO is also involved in the occurrence of tissue damage and inflammation in chronic inflammatory diseases (143). Rensen et al. reported that the number of neutrophils was increased in the liver of NASH patients than in those with fatty liver, and the enhanced inflammation observed in NASH patients was associated with increased expression and activity of MPO (130). The plasma MPO levels and number of MPO-positive cells in the liver were increased in patients with NASH (130). In addition, NASH patients showed an increase in the accumulation of proteins modified by hypochlorite and nitrates which can be formed by the MPO-H_2_O_2_ system (130). An increased population of MPO-positive cells was also associated with the upregulation of CXC chemokines and hepatic neutrophil infiltration in the liver of NASH patients (130).

The contribution of MPO to fatty liver to NASH progression was further clarified by another study conducted by Rensen et al. (132). In this study, NASH was induced by feeding LDLR-deficient mice with an HFD. LDLR-null mice with MPO deficiency in the hematopoietic system showed reduced inflammation and fibrosis in the liver, indicating that neutrophil-derived MPO plays a crucial role in the development of NASH in mice. Furthermore, MPO-deficient mice showed a reduction in hepatic cholesterol, which is known to exacerbate NASH progression.

Feeding mice with a methionine and choline-deficient diet (MCD) is a classic method of inducing NASH in mice (90, 144). MCD-induced NASH is accompanied by an increase in MPO expression by neutrophils in the liver (131). Whole-body deletion of the MPO gene attenuates hepatocyte death and the severity of NASH and fibrosis (131). Mechanistically, MPO-derived oxidative stress causes hepatocyte injury through mitochondrial permeability transition and activates HSCs, which results from the crosstalk between neutrophilic MPO, hepatocytes, and HSCs (131).

MPO deficiency could also prevent the development of NASH in mice by attenuating liver injury and fibrosis induced by feeding a high-fat, high-cholesterol, high-carbohydrate diet (91), a widely used dietary model of NASH with a combination of different types of toxic substances (145). This study also reported that pharmacological inhibition of MPO through treatment with AZM198, an MPO inhibitor, could repress NASH progression and liver fibrosis induced by feeding a high-fat, high-cholesterol diet (HFHCD) (91).

#### 3.3.2 Neutrophil Elastase

Neutrophil elastase (NE) is a serine protease that is released by neutrophils during inflammation through the degranulation process (146). NE is also important in NET formation because it contributes to the histone degradation and chromatin decondensation during NET formation (147, 148). NE is not only implicated in pathogen infection but also in sterile inflammation, which commonly develops in the liver of NASH patients (126).

NE that is released into the extracellular space is bound to alpha-1-antitrypsin (AAT), which inhibits the activity of NE; thus, the ratio of NE to AAT in the serum is used to predict the activity of NE (149–151). In a study that recruited NAFLD patients and healthy controls, the ratio of NE to AAT was higher in NAFLD patients and was closely associated with liver inflammation in patients with NASH, indicating that it can be used as a marker to evaluate the severity of NASH in humans (133). Another clinical study reported that the plasma concentration of NE was associated with the severity of NAFLD. The advanced stages of NAFLD with NASH and fibrosis features are characterized by higher levels of hepatic NE (152). This study also showed that the hepatic levels of proteinase 3, another type of neutrophil serine protease, were correlated with the severity of NAFLD (152, 153).

Talukdar et al. demonstrated that HFD feeding increased the infiltration of neutrophils in the liver of mice, and liver-infiltrating neutrophils inhibited the insulin signaling by degradation of insulin receptor substrate 1 (141). They also showed that NE possibly mediated these effects of neutrophils on liver metabolism because deletion of the *Elane* gene, which encodes NE, increased the hepatic insulin sensitivity and reduced the expression of hepatic inflammatory genes in HFD-fed mice (141). *Elane*−/− mice were also resistant to western diet-induced NASH, with decreased hepatic expression of lipogenic and inflammatory genes and reduced hepatic levels of ceramides that promote inflammation through a variety of signaling pathways (134). The pharmacological inhibition of NE by treatment with sivelestat reduced the serum transaminase activity, expression of inflammatory mediators, and NASH score in *Apoe*-deficient mice fed with an HFHCD that showed NASH phenotypes (142). This study also showed that the MCD-induced NASH was prevented by the Ly6G monoclonal antibody-induced depletion of neutrophils (142).

Although previous studies have suggested that NE contributes to the pathogenesis of NAFLD, our recent data revealed that deletion of the *Elane* gene did not affect the serum ALT levels in HFD^+CXCL1^-fed mice (16). Conflicts among these data may be because of the different models and wild-type control mice used (littermate controls were used in our studies).

#### 3.3.3 Lipocalin 2

Lipocalin 2 (LCN2) is a proinflammatory cytokine found in specific granules of neutrophils. LCN2 has biological functions in various processes, ranging from innate immunity to cell death, cell proliferation, and metabolism (154–158). LCN2 has been implicated in the development of inflammatory and metabolic diseases (159, 160) and is generally induced by injury and inflammatory conditions in the liver (161). The LCN2 levels are elevated in the experimental models of NASH and promote inflammation by attracting neutrophils and inducing the expression of proinflammatory cytokines (e.g., TNF-α, IL-1β, MCP-1) (135, 162). In addition, LCN2 was found to be elevated in the livers of NASH patients. The mRNA and protein levels of LCN2 in the liver were higher in NASH patients than in those with fatty liver (136). This increase is thought to be because of the proinflammatory environment in NASH, as evidenced by the upregulation of LCN2 in the HepG2 hepatoma cell line upon exposure to proinflammatory mediators, such as TNF-α and IL-6 (136). The expression of LCN2 in the liver was highly increased in fatty liver Shionogi (FLS) mouse model (138) which features spontaneous development of fatty liver that progresses to NASH and eventually hepatocellular carcinoma (163, 164). In the FLS model, CXCL1 was found to be elevated, which might further exacerbate the progression of NASH in concert with LCN2 (138). In line with these studies, Xu et al. reported that LCN2 mRNA levels were increased by more than 28-folds in a high-fat, high-sugar-induced NASH mouse model; their protein-protein interaction analysis using Search Tool for the Retrieval of Interacting Genes (STRING, http://string-db.org) database supported the notion that LCN2 might physically interact with various inflammatory mediators (e.g., IL-1R2, IL-3, IL-6, IL-18, and IL-17A) or might be involved in the inflammatory cascades mediated by inflammatory mediators (137). HFHCD-induced NASH was accompanied by an upregulation of LCN2 expression in the liver, in which the major LCN2-enriched cell type was neutrophils (135). Genetic ablation of LCN2 in *Apoe*-null mice significantly suppressed the severity of hepatic injury and inflammation, whereas chronic administration of recombinant LCN2 enhanced the severity of HFHCD-induced NASH (135). Mechanistically, LCN2 promoted the activation of C-X-C motif chemokine receptor 2-mediated extracellular signal-regulated kinase (ERK), which enhances the crosstalk between neutrophils and macrophages in the liver and induces inflammatory responses (135).

#### 3.3.4 Potential Roles of Neutrophil-Derived S100A8/A9 in NASH Development

S100A8 and S100A9 are Ca^2+^-binding proteins that belong to the S100 family and account for a substantial proportion of the neutrophil cytosolic protein population (165–167). S100A8 and S100A9 preferentially form heterodimers; the S100A8/A9 heterodimer is released by activated neutrophils and is implicated in the pathogenesis of various diseases as an innate immune mediator (168–170). The expression of S100A8 and S100A9 is elevated in the visceral adipose tissues of obese patients with diabetes (171). However, the contribution of S100A8/A9 to NASH development is not well understood although the serum levels of S100A8 and S100A9 are elevated in NASH patients (172). As it has been increasingly recognized that the crosstalk between the liver and adipose tissue plays a crucial role in NASH development, it is reasonable to further examine the role of adipose tissue proteins S100A8/A9 in NASH development. Indeed, S100A8 and S100A9 are upregulated in the adipose tissue samples of NASH patients and CXCL1-induced experimental NASH models, and the administration of paquinimod, a S100A9 inhibitor, attenuated the CXCL1-induced NASH in mice (128). This finding further supports the possibility that neutrophil-derived specific molecules might be implicated in the crosstalk between organs that exacerbates NASH.

### 3.4 Role of NET in NASH Development

NET is a web-like structure of DNA fibers composed of histones and granule proteins (173). NETs are formed by expulsion of the nuclear materials of neutrophils into the extracellular space (147). NET was originally reported to be a mechanism through which neutrophils participate in host defense by capturing and killing microorganisms and preventing their dissemination (174); however, increasing evidence has supported the role of NET as a critical mediator of sterile inflammation, which is critically implicated in the development of various diseases including cancer (175–177). NET also potentiates neutrophil function through a positive feedback loop and thus has attracted attention as a therapeutic target for inflammatory diseases (178).

NET formation is stimulated in experimental NASH models. In the Stelic animal model of NASH (STAM), neonatal streptozotocin treatment and HFD induces NASH and hepatocellular carcinoma in mice by creating a diabetic and obesogenic environment (179). STAM mice showed elevated neutrophil infiltration and NET formation in the liver (92). Inhibition of NET formation through deoxyribonuclease (DNase) treatment or the deletion of the gene encoding PAD4 attenuated the monocyte infiltration and inflammation in the liver of STAM mice, although steatosis was not significantly improved (92). In addition, DNase treatment or PAD4 deletion inhibited the NASH-associated hepatocellular carcinoma development in STAM mice. Moreover, the serum levels of MPO-DNA, which is a marker of NET, were elevated in NASH patients than in healthy individuals (92). A more recent study revealed that not only the serum marker but also the hepatic marker of NET formation (e.g., citrullinated histone H3) was increased in the liver of NASH patients and that NET was correlated with NAFLD severity (139).

NET formation was also promoted in a methionine-choline-deficient high-fat diet (MCDHFD)-induced NASH model. In mice fed with an MCDHFD, NETs were detected in the early stages of NASH, and the depletion of NETs by intraperitoneal injection of DNase I alleviated the MCDHFD-induced liver injury, inflammation, and fibrosis (140). During the development of MCDHFD-induced NASH, hepatic sphingosine 1-phosphate (S1P) levels were correlated with NET formation. S1P activated p38MAPK- and ERK-mediated ROS production in neutrophils *via* the S1P receptor 2 signaling pathway, which switched apoptosis to NETosis and promoted NASH development (140).

## 4 Potential Beneficial Functions of Neutrophils in NASH

Damaged or infected tissues undergo a series of inflammatory processes, where neutrophil infiltration is one of the first events to remove pathogenic microorganisms or overcome injuries. These inflammatory processes are usually followed by tissue repair; however, excessive and prolonged inflammation that is not properly resolved by various repair mechanisms may lead to chronic destructive inflammation and fibrosis, which are critically implicated in the development of various inflammatory diseases.

Resolution of inflammation is a coordinated and active process that is designed to maintain tissue homeostasis (180, 181). Timely resolution of inflammation is important because tissue integrity and function are impaired during inflammation; thus, prolonged inflammation may lead to collateral tissue damage (182, 183). Neutrophil infiltration is correlated with the severity of tissue damage and inflammation (184). Thus, neutrophil apoptosis and the clearance of apoptotic neutrophils are tightly regulated in order to resolve the inflammation (185, 186). Apoptotic neutrophils are normally cleared from the affected site by the macrophages through the process of efferocytosis (187).

Neutrophils are generally thought to initiate and aggravate inflammation; the current review has so far discussed the deleterious function of neutrophils, that is, exacerbation of tissue injury and inflammation during the pathogenesis of liver diseases. However, recent publications have increasingly described the pro-resolving and tissue-restorative functions of neutrophils (12, 188). A study by Wang et al. reported the cooperation of neutrophils with macrophages which promotes the conversion of proinflammatory monocytes to pro-resolving macrophages to orchestrate the resolution of tissue inflammation and repair (189). Proteases derived from neutrophils, such as NE, inhibit the production of IL-1β and TNF-α and cause the degradation of these cytokines (190). Neutrophils also inhibit the production of cytokines induced by lipopolysaccharides or the fragment of the cell wall of gram-positive bacteria (191). Although NETs promote inflammation, they capture and degrade proinflammatory mediators, especially in sites where the density of neutrophils is high, which is thought to be associated with the action of neutrophilic proteases that are components of NETs (192).

Formyl peptide receptor 2 binds multiple pro-resolving ligands, including annexin A1, lipoxin A4, 15-epi-lipoxin A5, and 17-epi-resolvin D1, which contribute to multiple processes that mediate the maintenance of tissue homeostasis under inflammatory conditions (193). Some of these processes include inhibition of neutrophil infiltration and attachment and stimulation of neutrophil apoptosis, thereby attenuating tissue injury and accelerating inflammation resolution (193). Interestingly, neutrophils produce and release some of these pro-resolving ligands (e.g., annexin A1) *via* the membrane-derived microvesicles, through which neutrophils can actively participate in the resolution of inflammation (194–197).

Researchers in the field of hepatology have also recently demonstrated the pro-resolving role of neutrophils in several models of liver diseases, including NASH. Calvente et al. reported that depletion of neutrophils in mice recovering from MCD-induced NASH or CCl_4_-induced fibrosis prolonged the period of tissue damage, inflammation, and fibrosis (198). Mechanistically, neutrophils participate in the resolution of inflammation by transferring miR-223 from neutrophils to macrophages *via* the neutrophil-derived EVs or as a complex with lipoproteins or argonaut 2 (198). In macrophages, miR-223 inhibited the activity of NOD-, LRR-, and pyrin domain-containing protein 3 (NLRP3) inflammasomes and induced the polarization of restorative macrophages, which release cytokines such as IL-10, leading to the resolution of inflammation and fibrosis. The anti-inflammatory role of miR-223 was also observed in a study by He et al.; they showed that miR-223-deficient mice were more vulnerable to diet-induced NASH and hepatocellular carcinoma (109). It was later demonstrated that neutrophil-derived EVs deliver miR-223 to hepatocytes where they exert their anti-inflammatory and anti-tumorigenic functions (109, 118).

Advancements in single-cell analysis have made it possible to study the function of different subsets of immune cells in the development of various diseases (199). In particular, recent publications have elucidated several distinct subsets of macrophages that are enriched in the livers of humans and mice with NASH (65, 67). However, neutrophils have been traditionally thought to be homogeneous, and little is known about the different subsets of neutrophils that may function distinctively during the development of NASH. The notion that neutrophils are both proinflammatory and pro-resolving warrants further investigation of the heterogeneity of neutrophils in the context of NASH development.

## 5 Neutrophils as Potential Therapeutic Targets for the Treatment of NASH

The involvement of neutrophils in multiple processes in the pathogenesis of liver diseases has made them attractive targets for therapeutic intervention. As discussed in this review, neutrophils generally exacerbate inflammation and liver injury, which have established strategies to deplete neutrophils or inhibit the activity of neutrophils. Blockade of granulocyte colony-stimulating factor 3 receptor with monoclonal antibodies inhibits the production and activation of neutrophils and has been suggested to ameliorate several diseases, including arthritis (200, 201). Neutrophils are short lived and susceptible to apoptosis, and deletion of the *Foxo3a* gene that is needed for neutrophils to survive prevented inflammatory diseases in mice due to excessive neutrophil apoptosis (202). However, whether pharmacological interventions to reduce the neutrophil population in the circulation or at the site of inflammation are useful for treating NASH should be investigated further.

Because proteins expressed by neutrophil granules significantly contribute to the pathogenic functions of neutrophils, researchers have attempted to develop pharmacological modulators that can control the activities of granule proteins, such as NE, MPO, and LCN2. Elafin and AAT are endogenous factors that have the ability to inhibit NE, and emerging evidence has demonstrated that they may attenuate several diseases in experimental models (203, 204). Elafin is an antimicrobial and anti-inflammatory protein with a molecular weight of approximately 6 kDa (205). Lentiviral overexpression of elafin inhibits HFD-induced steatosis in mice (206). MPO has also been studied as a target of anti-neutrophil therapies because it mediates multiple steps in neutrophil-induced inflammation and tissue damage (207). It has been attempted to inhibit the activity of MPO using chemicals that block the active site of MPO, remove MPO from the chlorination reactions, or scavenge HOCl; however, whether these strategies are valid for the treatment of NASH in experimental animal models remain unknown (208–214). The therapeutic potential of LCN2 inhibition has been examined for the treatment of various diseases, including cancer (215, 216), which has led to the investigation of biological therapeutics such as monoclonal antibodies, RNA interference technology, and nanoparticle-based LCN2 modulators (158, 161, 217–219).

NET formation was originally highlighted as an immune response of neutrophils against microorganism infection; however, accumulating evidence has suggested the involvement of NETs in sterile inflammation, which has facilitated the investigation of the potential application of anti-NETosis therapy in the treatment of inflammatory diseases (147). PAD4 is regarded as one of the most important enzymes involved in the formation of NETs (220), and deletion of the gene that encodes PAD4 or pharmacological inhibition of PAD4 has been widely implemented to study the pathogenic role and therapeutic potential of NETs (221). The study by Tsung et al. on the role of NET in the development of NASH-associated hepatocellular carcinoma corroborates the applicability of anti-NETosis therapy in the treatment of full-spectrum NAFLD (92).

Neutrophils have been actively studied as targets for pharmacological intervention in multiple diseases including autoimmune diseases, atherosclerosis, asthma, infectious diseases, psoriasis, and sepsis (222). Either enhancement or inhibition of the function of neutrophils has been studied as the strategic approach against these diseases. For example, blocking neutrophil recruitment, blocking neutrophil-derived mediators, and targeting NETs have been attempted to inhibit the excessive tissue damage caused by neutrophils in pulmonary diseases, atherosclerosis, and psoriasis (125). However, little is known about whether these strategies can produce positive outcomes for the treatment of NASH. Considering the involvement of neutrophils in the multiple processes of NASH pathogenesis that have been discussed in this review, it is justifiable to further explore the feasibility of anti-neutrophil therapy as a therapeutic strategy for NASH.

## 6 Conclusions

Knowledge on the role of neutrophils has rapidly expanded in recent years, which has not only enhanced our understanding of the pathogenic mechanism of NASH, but also laid the foundation for its application in establishing an experimental NASH model that may be utilized for drug screening. Although the clinical data to support the applicability of the neutrophil-modulating approach have yet to be presented, the potential benefit of this strategy has been increasingly supported by studies conducted in NASH animal models. Moreover, the population of neutrophils can be more heterogeneous than we currently recognize. Advances in analytical techniques, such as the advent of single-cell RNA sequencing, have further raised questions regarding the different subsets of neutrophils that may distinctively participate in NASH pathogenesis. Elucidation of these remaining questions will help us better elucidate the role of neutrophils in NASH development and open new avenues for the therapeutic intervention of NASH.

## Author Contributions

SH, HY, and SM wrote the manuscript. YC edited the manuscript and figure. BG supervised the writing and edited the manuscript. All authors contributed to the article and approved the submitted version.

## Funding

This work was supported by Pusan National University Research Grant, 2021 (SH). This work was also supported by the National Research Foundation of Korea (NRF) grant funded by the Korea government (MSIT) (No. 2021R1F1A1056033) (SH) and by the intramural program of NIAAA, NIH (BG).

## Conflict of Interest

The authors declare that the research was conducted in the absence of any commercial or financial relationships that could be construed as a potential conflict of interest.

## Publisher’s Note

All claims expressed in this article are solely those of the authors and do not necessarily represent those of their affiliated organizations, or those of the publisher, the editors and the reviewers. Any product that may be evaluated in this article, or claim that may be made by its manufacturer, is not guaranteed or endorsed by the publisher.
